# Connaissances, attitudes et pratiques des élèves adolescentes sur l’hygiène menstruelle à Kisangani

**DOI:** 10.11604/pamj.2025.50.44.44917

**Published:** 2025-02-07

**Authors:** Alpha Ngandru Kiza, Samuel Bosongo Itigaino, Gédéon Katenga Bosunga, Charles Kayembe Tshilumba, Alliance Tagoto Tepunipage, Eugène Basandja Longembe, Santhos Lobwa Bosongo, Jean Didier Bosenge Nguma, Joris Losimba Likwela, John Panda Lukongo Kitronza

**Affiliations:** 1 Département de Santé Publique, Faculté de Médecine et de Pharmacie, Université de Kisangani, Kisangani, République Démocratique du Congo,; 2 Département de Gynécologie-Obstétrique, Faculté de Médecine et de Pharmacie, Université de Kisangani, Kisangani, République Démocratique du Congo,; 3 Département de Médecine Interne, Faculté de Médecine et de Pharmacie, Université de Kisangani, Kisangani, République Démocratique du Congo,; 4 Département de Santé Publique, Université Officielle de Yabaondo, Yabaondo, République Démocratique du Congo

**Keywords:** Connaissances, attitudes, pratiques, hygiène menstruelle, adolescentes, écoles secondaires, Kisangani, Knowledge, attitudes, practices, menstrual hygiene, adolescent girls, secondary schools, Kisangani

## Abstract

**Introduction:**

la menstruation est un phénomène physiologique qui s'accompagne des besoins cruciaux pour sa bonne gestion. Elle est entourée de silence, mythes et tabous, d'où la nécessité d'évaluer le niveau de connaissances, les attitudes et les pratiques des élèves adolescentes sur l'hygiène menstruelle (HM), les facteurs qui y sont associés, ainsi que les répercussions sur leur scolarité.

**Méthodes:**

une étude transversale analytique a été réalisée de septembre 2023 à février 2024 auprès de 553 élèves adolescentes issues de 38 écoles secondaires de Kisangani. Les données recueillies via un questionnaire structuré, ont été analysées avec Stata 13. L'analyse univariée a décrit les caractéristiques sociodémographiques et les connaissances, attitudes et pratiques des participantes. Des analyses multivariées ont identifié les facteurs associés à la bonne connaissance sur la menstruation et une bonne gestion de l'hygiène menstruelle, en utilisant les odds ratios ajustés pour apprécier les associations.

**Résultats:**

la majorité des participantes, âgées de 15 à 17 ans avec un âge moyen à la ménarche était de 12,8 ans. Elles étaient inscrites dans des écoles privées et provenaient de familles de niveau socio-économique moyen. Alors que 70,2% avaient suivi un cours sur l'hygiène menstruelle et que 91,5% avaient reçu des informations sur les menstruations avant leurs premières règles, seulement 11,0% avaient de bonnes connaissances sur l'hygiène menstruelle et la prévalence de bonnes pratiques de l'hygiène menstruelle n'était que 12,3%. L'analyse multivariée a montré qu'avoir sa résidence dans la commune de Makiso (ORa= 1,8; IC95%= 1,0-3,4; p= 0,049), suivre un cours de l'éducation à la vie qui aborde l'hygiène menstruelle (ORa= 2,7; IC95%= 1,1-6,3; p= 0,025), être en terminale (ORa= 2,4; IC95%= 1,1-5,3; p= 0,025) et avoir comme sources d'information des mères ou des soeurs (ORa= 2,7; IC95%=1,4-5,1; p= 0,002) étaient significativement associés à la bonne connaissance sur les menstruations et l'HM. De même, le fait d'être inscrites à l'école privée (ORa=0,6 ; IC95%=0,3-0,9 ; p=0,041) et le niveau d'étude universitaire des mères (ORa=1,9 ; IC95%= 1,1-3,3; p= 0,016) a montré une association avec la bonne pratique.

**Conclusion:**

bien que la plupart des adolescentes aient été informées des menstruations avant la ménarche, seulement 11% possèdent une bonne connaissance de l'hygiène menstruelle et 12,3% adoptent de bonnes pratiques. L'éducation à la vie, en particulier sur l'hygiène menstruelle, est cruciale pour améliorer ces aspects.

## Abstract (en)

**Introduction:**

menstruation is a physiological phenomenon that is accompanied by crucial needs for proper management. It is shrouded in silence, myth and taboo, hence the need to assess the level of knowledge, attitudes and practices of adolescent students about menstrual hygiene (MH), factors associated with it and repercussions on their schooling.

**Methods:**

we conducted an analytical cross-sectional study on 553 adolescent girls from 38 secondary schools in Kisangani City between September 2023 and February 2024. Data were collected using a structured questionnaire and analyzed using Stata 13 software. Univariate analysis summarized the sociodemographic characteristics of the participants, as well as their knowledge, attitudes, and practices regarding menstrual hygiene. Multivariate analyses identified factors associated with good knowledge of menstruation and menstrual hygiene (MH), as well as good menstrual hygiene management, using adjusted odds ratios to assess the associations.

**Results:**

the majority of participants, aged 15 to 17 years, with an average age of menarche of 12.8 years, were enrolled in private schools and came from families with a middle socio-economic status. While 70.2% had attended a course on menstrual hygiene and 91.5% had received information about menstruation before their first period, only 11.0% had good knowledge of menstrual hygiene, and the prevalence of good menstrual hygiene practices was only 12.3%. Multivariate analysis showed that living in the Makiso commune (ORa=1.8; 95% CI=1.0-3.4; p=0.049), attending a family life education course that covers menstrual hygiene (ORa=2.7; 95% CI=1.1-6.3; p=0.025), being in the final year of secondary school (ORa=2.4; 95% CI=1.1-5.3; p=0.025), and having mothers or sisters as sources of information (ORa=2.7; 95% CI=1.4-5.1; p=0.002) were significantly associated with good knowledge of menstruation and menstrual hygiene. Furthermore, attending a private school (ORa=0.6; 95% CI=0.3-0.9; p=0.041) and having a mother with a university education (ORa=1.9; 95% CI=1.1-3.3; p=0.016) were associated with good practices.

**Conclusion:**

although most adolescents had been informed about menstruation before menarche, only 11% had good knowledge of menstrual hygiene and 12.3% adopted good practices. Life education, particularly on menstrual hygiene, is crucial to improving these aspects.

## Introduction

Le développement humain chez la femme inclut des transformations majeures, telles que la puberté et la ménarche, marquant le début du cycle menstruel et de la vie reproductive. La menstruation génère des besoins spécifiques en matière d'hygiène menstruelle (HM), incluant l'accès aux informations adéquates, produits absorbants appropriés et installations sanitaires privées, qui sont des droits fondamentaux [1-3]. La gestion de l'HM est essentielle à la santé globale et nécessite une approche systématique dans les politiques de santé publique [2].

Le contexte socio-culturel, la pénurie en ressources et les facteurs physiques influencent considérablement l'expérience menstruelle, un problème particulièrement important dans les pays à revenu moyen et faible, dont ceux d'Afrique subsaharienne où la gestion de l'HM demeure un défi de santé publique majeur [4,5]. En Égypte et en Ouganda, des études ont montré que les pratiques de gestion de l'HM sont insuffisantes et souvent inappropriées, avec des filles ne satisfaisant pas aux critères d'une bonne gestion, notamment en matière de changement des produits absorbants et d'élimination des déchets [2,6,7]. Les pratiques inadéquates augmentent les risques d'infections et de stress psychosocial, et contribuent à l'absentéisme scolaire [1-3].

En République démocratique du Congo (RDC), la mauvaise connaissance sur les menstruations persiste parmi les filles et les garçons. Environ 69,2% des filles utilisent des produits absorbants inappropriés, en raison du manque d'informations, du coût et de l'indisponibilité des produits d'hygiène. Dans la ville de Kisangani, une étude menée auprès des étudiantes de l'université de Kisangani a révélé que près de 57,1% d'entre elles rencontraient des difficultés pour gérer leurs menstruations, et 56,0% interrompaient leurs activités à cause de leurs règles, et 26,9% éliminaient de façon adéquate les produits absorbants dans les toilettes [8]. De plus, des croyances associées à la menstruation persistaient chez 20,0% des étudiantes [9].

Si la gestion de l'HM n'était pas optimale chez les étudiantes de l'université, la situation des élèves adolescentes des écoles secondaires pourrait être encore plus problématique. À Kisangani, peu d'études existent sur la gestion de l'HM chez les élèves adolescentes, d'où la nécessité d'évaluer le niveau de connaissances, les attitudes et les pratiques des élèves adolescentes en matière d'HM, les facteurs qui y sont associés ainsi que les répercussions sur leur scolarité.

## Méthodes

### Design et milieu de l´étude

Une étude transversale analytique a été réalisée du 15 septembre 2023 au 15 février 2024 dans les écoles secondaires de la ville de Kisangani, capitale de la province de la Tshopo, située au nord-est de la République démocratique du Congo. Le taux d'alphabétisation dans cette région est de 47,4% pour les niveaux secondaire et supérieur, tandis que le taux de scolarisation des filles atteint 73% [10].

### Population d´étude

La population de l'étude était constituée des élèves filles des écoles secondaires de la ville Kisangani qui comprend 228 écoles secondaires dont 140 publiques et 88 privées. Les critères d'inclusion étaient: être fille âgée de 12 ans ou plus, avoir déjà connu la ménarche, être inscrite dans une école sélectionnée, être présente lors de la collecte des données et avoir consenti à participer. Les élèves éligibles absentes le jour de la collecte des données ou n'ayant pas consenti ont été exclues. La taille minimale de l'échantillon, calculée à partir d'une prévalence estimée de 60% pour les bonnes pratiques d'HM, avec une précision de 5% et un taux de non-réponse de 20%, était de 553 élèves filles. Les données ont été recueillies dans 38 écoles, sélectionnées selon la méthode de *Lot Quality Assessment Survey* (*LQAS*), choisi pour sa rapidité et une alternative fiable aux autres méthodes qui exigent un grand échantillon [11]. L'échantillonnage a été réalisé à deux niveaux. Au premier niveau, un échantillonnage systématique de 19 écoles publiques et 19 écoles privées, basé sur la liste des écoles secondaires de la ville de Kisangani fournies par la direction provinciale de l'enseignement primaire, secondaire et technique. Nous avons ensuite procédé à un échantillonnage systématique de 19 écoles dans chaque groupe. Au deuxième niveau, nous avons calculé les proportions des filles de chaque école sélectionnée, en divisant le nombre de filles de chaque école par le total des filles de 38 écoles sélectionnées; ensuite, une répartition proportionnelle du nombre de filles par école en fonction de leur poids relatif dans l'effectif total. Au sein des écoles, les filles consentantes ont été sélectionnées dans chaque classe, constituant un échantillon final de 587 élèves.

### Collecte des données

Les données ont été recueillies à l'aide d'un questionnaire pré-testé par des enquêtrices sages-femmes formées et supervisées par le premier auteur. Ce questionnaire portait sur les caractéristiques sociodémographiques, les caractéristiques des menstruations, les connaissances, attitudes et pratiques de l'HM, ainsi que l'impact des menstruations sur la scolarité. Avant la collecte, un briefing a été organisé avec les adolescentes consentantes pour leur expliquer le contenu.

### Définition des variables

Les variables étudiées incluaient: 1) les caractéristiques sociodémographiques tels que l´âge, la religion, le niveau d´instruction et l´occupation de la mère, le niveau socio-économique des élèves; 2) les caractéristiques des menstruations dont l'âge de la ménarche, la durée des cycles menstruels, leur régularité et la quantité du flux sanguin; 3) les connaissances sur les menstruations, notamment les informations sur les menstruations avant la ménarche, les connaissances sur le cycle menstruel, les sources d´information avant la ménarche, les causes de menstruation, origine du sang; 4) les attitudes et pratiques de l´HM, à savoir les réactions aux premières menstruations, le type de produit absorbant utilisé, la fréquence de changement du produit absorbant, la méthode d´élimination du produit absorbant, le lieu de séchage du produit absorbant, le nettoyage des parties génitales pendant les menstruations, le changement du produit absorbant à l´école.

Deux variables dépendantes ont été évaluées: la prévalence de bonne connaissance de la menstruation et de l'HM, ainsi que la prévalence de bonnes pratiques de l'HM. La première a été calculée par le rapport entre le nombre de filles qui remplissaient tous les critères de bonne connaissance de menstruation et de l'HM et le nombre total des filles incluses dans l'étude. Ces critères ont été définis par l'équipe de recherche et comprenaient la connaissance des origines des menstruations, des causes des menstruations et des types du matériel absorbant approprié. La seconde qui était basée sur les critères de bonnes pratiques de l'HM définis par Hennegan *et al*. [7], comprenaient le type du matériel absorbant utilisé, la fréquence de son changement, le lavage de l'appareil génital et l'existence des toilettes garantissant l'intimité nécessaire pour bain intime et change des tissus absorbants.

### Analyses statistiques

Après nettoyage des questionnaires (n=587), 34 questionnaires (6%) ont été jugés non valides en raison de réponses manquantes, laissant un échantillon de 553 questionnaires pour l'analyse. Les données ont été saisies simultanément par deux opérateurs dans Excel, puis traitées avec le logiciel STATA 13. L'analyse univariée a permis de décrire les caractéristiques de l'échantillon, les connaissances, attitudes et pratiques liées à l'HM ainsi que de calculer la prévalence des bonnes connaissances et pratiques de l'HM. Pour analyser les facteurs associés à la bonne connaissance et aux bonnes pratiques de l'HM, nous avons procédé à l'analyse bivariée en utilisant les Odds Ratio (OR) avec un intervalle de confiance de 95%. Les variables ayant montré des associations significatives ont été incluses dans l'analyse multivariée par régression logistique, avec une approche pas à pas au seuil de 5%. Les OR ajustés et les p-values de Wald ont été présentés.

### Considérations éthiques

Avant la collecte des données, les autorisations nécessaires ont été obtenues du Directeur Provincial de l'EPST de la Tshopo ainsi que des responsables des établissements scolaires concernés. Un consentement éclairé a été recueilli auprès des parents des adolescentes, qui ont été informés des objectifs de l'étude, tout en garantissant la confidentialité et l'anonymat des données collectées. La participation était strictement volontaire, et les participantes pouvaient se retirer de l'étude à tout moment sans aucune conséquence.

## Résultats

### Caractéristiques sociodémographiques

La majorité des participantes étaient inscrites dans des écoles privées (54,6%), avait un âge moyen de 15 ans, et provenaient majoritairement de familles de niveau socio-économique moyen (75,8%). Plus de la moitié priaient dans des églises de réveil (54,4%). En termes de résidence, les élèves des écoles publiques étaient davantage concentrées à Kabondo (13,9%) et Lubunga (10,0%), tandis que celles des écoles privées résidaient principalement à Mangobo et Makiso. Par ailleurs, plus des deux tiers des participantes (70,2%) ont suivi des cours sur l'hygiène menstruelle, avec une légère prédominance dans les écoles privées (71,9%) par rapport aux écoles publiques (68,1%) (Tableau 1).

**Tableau 1:** caractéristiques sociodémographiques des enquêtées

Variables	Privé n=302 (%)	Publique n=251 (%)	Total n=553 (%)
**Age (Moyenne ± SD)**	**15±1,8**	**15±1,8**	**15±1,8**
12 à 14 ans	109 (36,1)	95 (37,9)	204 (36,9)
15 à 17 ans	132 (43,7)	102 (40,6)	234 (42,3)
≥ 18 ans	61 (20,2)	54 (21,5)	115 (20,8)
**Commune de résidence**			
Kabondo	10 (3,3)	35 (13,9)	45 (8,1)
Kisangani	18 (6,0)	13 (5,2)	31 (5,6)
Lubunga	2 (0,7)	25 (10,0)	27 (5,0)
Makiso	87 (28,8)	55 (21,9)	142 (25,7)
Mangobo	114 (37,8)	59 (23,5)	173 (31,3)
Tshopo	71 (23,5)	64 (25,5)	135 (24,4)
**Religion**			
Catholique	49 (16,2)	59 (23,5)	108 (19,5)
Eglises de réveil	179 (58,9)	123 (49,0)	301 (54,4)
Kimbanguiste	15 (5,0)	14 (5,6)	29 (5,2)
Musulmane	17 (5,6)	15 (6,0)	32 (5,8)
Protestante	43 (14,2)	40 (16,0)	83 (15,0)
**Niveau d’étude**			
Enseignement de base (8^e^)	80 (26,5)	84 (33,5)	164 (29,7)
Moyen (1^re^ et 2^e^ humanités)	137 (45,4)	111 (44,2)	248 (44,8)
Terminal (3^e^ et 4^e^ humanités)	85 (28,1)	56 (22,3)	141 (25,5)
**Niveau socio-économique**			
Bas	44 (14,6)	33 (13,2)	77 (13,9)
Moyen	225 (74,5)	194 (77,3)	419 (75,8)
Elevé	33 (10,9)	24 (9,6)	57 (10,3)
Participation au cours d’éducation abordant l’HM*			
Oui	217 (71,9)	171 (68,1)	388 (70,2)
Non	85 (28,2)	80 (31,9)	165 (29,8)

* HM=hygiène menstruelle

### Caractéristiques des menstruations

L'âge moyen à la ménarche était de 12,8 ± 1,1 ans, sans différence notable entre les élèves des écoles privées (12,8 ± 1,1 ans) et publiques (12,9 ± 1,2 ans). Une durée de menstruations de 3 à 4 jours était prédominante (71,3%), avec une proportion similaire dans les écoles privées (71,5%) et publiques (70,9%). Les intervalles entre menstruations de 25 à 28 jours étaient majoritaires (53,8%). Enfin, la majorité des participantes utilisaient 3 à 4 garnitures par jour (55,5%) et les menstruations abondantes étaient davantage signalées dans les écoles publiques (16,7%) que dans les privées (10,3%) (Tableau 2).

**Tableau 2:** caractéristiques des menstruations

Variables	Privé n=302 (%)	Publique n=251 (%)	Total n=553 (%)
**Ménarche (ans) (Moyenne ± SD)**	**12,8±1,1**	**12,9±1,2**	**12,8±1,1**
**Durée des menstruations (jours)**			
1 à 2	24 (8,0)	12 (4,8)	36 (6,5)
3 à 4	216 (71,5)	178 (70,9)	394 (71,3)
≥5	62 (20,5)	61 (24,3)	123 (22,2)
**Intervalle entre les menstruations (jours)**			
<25	83 (28,8)	57 (23,6)	140 (26,4)
25 à 28	153 (53,1)	132 (54,6)	285 (53,8)
≥29	52 (18,1)	55 (21,9)	107 (19,8)
**Régularité des menstruations**			
Oui	280 (92,7)	217 (86,5)	497 (89,9)
Non	22 (7,3)	34 (13,6)	56 (10,1)
**Quantité des menstruations garnitures/jr)**			
Peu abondante (1 ou 2)	100 (33,1)	73 (29,1)	173 (31,3)
Modérée (3 ou 4)	171 (56,6)	136 (54,2)	307 (55,5)
Abondante (plus de 4)	31 (10,3)	42 (16,7)	73 (13,0)

### Connaissances sur les menstruations

La majorité des élèves des écoles privées (90,4%) et publiques (92,8%) étaient informées sur les menstruations, principalement par leurs mères (63,6%). Environ 26,9% des adolescentes connaissaient la définition du cycle menstruel, et 41,8% les considéraient comme un phénomène physiologique. Quatre-vingt-sept virgule deux pour cent, savaient quel type de tissu absorbant utiliser. Toutefois, seulement 11,0% avaient de bonnes connaissances sur l'hygiène menstruelle et la menstruation, sans différences notables entre les écoles privées et publiques (Tableau 3).

**Tableau 3:** connaissances sur les menstruations

Variables	Privé n=302 (%)	Publique n=251 (%)	Total n=553 (%)
**Informées sur les menstruations avant la ménarche**			
Oui	273 (90,4)	233 (92,8)	506 (91,5)
Non	29 (9,6)	18 (7,2)	47 (8,5)
**Si oui, sources d’information**	**n=273 (%)**	**n=233 (%)**	**n=506 (%)**
Mères	183 (67,0)	139 (59,7)	324(63,6)
Sœurs	44 (16,1)	39 (16,7)	83(16,4)
Amies	22 (8,1)	29 (12,5)	51(10,1)
Enseignants	14 (5,1)	12 (5,2)	26(5,1)
Églises	0 (0,0)	3 (1,3)	3(0,6)
Médias	1 (0,4)	0 (0,0)	1(0,2)
Autres	9 (3,3)	11 (3,46)	20(3,17)
**Définition de cycle menstruelle**			
Absence des menstrues	43 (14,2)	32 (12,8)	75 (13,6)
Présence des menstrues	99 (32,8)	95 (37,9)	194 (35,1)
Début des menstruations et veille de l’autre règle	97 (32,1)	52 (20,7)	149 (26,9)
Aucune idée	63 (20,9)	72 (28,7)	135 (24,4)
**Cause des menstruations**			
Maladies des femmes	80 (26,5)	82 (32,7)	162 (29,3)
Malédictions de Dieu	27 (8,9)	9 (3,6)	36 (6)
Phénomène physiologique	121 (40,1)	110 (43,8)	231 (41,8)
Aucune idée	74 (24,5)	50 (19,9)	124 (22,4)
**Origine des menstruations**			
Appareil urinaire	8 (2,7)	15 (6,0)	23 (4,2)
Matrice ou utérus	60 (19,9)	57 (22,7)	117 (21,2)
Vagin	126 (41,7)	100 (39,8)	226 (40,9)
Aucune idée	108 (35,8)	79 (31,5)	187 (33,8)
**Type de serviette à utiliser**			
Bande hygiénique à usage unique	185 (61,3)	144 (57,4)	329 (59,5)
Coupe menstruelle	11 (3,7)	12 (4,8)	23 (4,2)
Linge propre réutilisable	83 (27,5)	70 (27,9)	153 (27,7)
Coton, papiers hygiéniques, pièces déchirées et sous-vêtement seul	23 (7,5)	25 (9,9)	48 (8,6)
**Bonne connaissance de menstruation et HM**			
Oui	33 (10,9)	28 (11,2)	61 (11,0)
Non	269 (89,1)	223 (88,8)	492 (89,0)

### Attitudes et pratiques

La majorité des élèves, qu'elles soient dans des écoles privées ou publiques, ont exprimé une attitude défavorable lors de leur première menstruation (81,6 %). Concernant la gestion menstruelle, la plupart utilisaient des bandes hygiéniques jetables (52,4 %) et changeaient leurs absorbants moins de trois fois par jour (58,8 %). La majorité privilégiait l'eau et le savon pour nettoyer les linges réutilisables (81,6 %) et séchaient ces derniers à l'extérieur au soleil (51,7 %). La prévalence de bonnes pratiques de l'HM était estimée 12,3 % (Tableau 4).

**Tableau 4:** attitudes et pratiques

Variables	Privée n=302 (%)	Publique n=251 (%)	Total n=553 (%)
**Attitude lors de la première règle**			
Favorable (calme)	50 (16,6)	53 (20,7)	102 (18,4)
Défavorable (choquée, dégoutée, effrayée, inquiète et honteuse)	252 (84,4)	199 (79,3)	451 (81,6)
**Type d’absorbant utilisé**			
Bande hygiénique à usage unique	157 (52,0)	133 (52,9)	293 (52,4)
Coton	2 (0,7)	3 (1,2)	5 (1,0)
Linge propre réutilisable	100 (33,1)	71 (28,3)	171 (31,0)
Papiers hygiéniques	9 (3,0)	7 (2,8)	16 (2,8)
Pièces déchirées d’une étoffe	7 (2,3)	8 (3,2)	15 (2,7)
Sous-vêtement seul	27 (8,9)	30 (11,6)	56 (10,1)
**Fréquence de changement d’absorbant**			
Moins de trois fois	180 (59,6)	145 (57,8)	325 (58,8)
Trois fois ou plus	122 (40,4)	106 (42,2)	228 (41,2)
**Produit de nettoyage de linge réutilisable**			
Eau	58 (19,2)	44 (17,5)	102 (18,4)
Eau et savon (antiseptique)	244 (80,8)	207 (82,5)	451 (81,6)
**Lieu de séchage de linge réutilisable nettoyé**			
Extérieur au soleil	156 (51,7)	130 (51,8)	286 (51,7)
Extérieur pas au soleil	58 (19,2)	61 (24,3)	119 (21,5)
Intérieur de la maison	53 (17,6)	43 (17,1)	96 (17,4)
Autres	35 (11,5)	17 (6,8)	52 (9,4)
**Elimination d’absorbant à usage unique**			
Brûler	101 (33,4)	96 (38,3)	197 (35,6)
Enfouir	8 (2,7)	3 (1,2)	11 (2,0)
Jeter dans les latrines	134 (44,4)	88 (35,1)	222 (40,1)
Jeter dans la poubelle	31 (10,3)	24 (9,6)	55 (10,0)
Autres	28 (9,3)	40 (15,9)	68 (12,3)
**Bain intime pendant les menstruations**			
Moins de trois fois	76 (25,2)	72 (28,7)	148 (26,8)
Trois fois ou plus	226 (74,8)	179 (71,3)	405 (73,2)
**Produit utilisé pour la toilette intime**			
Eau	60 (19,9)	99 (39,4)	159 (28,8)
Eau et savon	242 (80,1)	152 (60,6)	394 (71,2)
**Intimité nécessaire pour changer d’absorbant**			
Oui	176 (58,3)	143 (57,0)	319 (57,7)
Non	126 (41,7)	108 (43,0)	234 (42,3)
**Bonne pratique de l’HM**			
Oui	45 (14,9)	23 (9,2)	68 (12,3)
Non	257 (85,1)	2 (91)	497 (88)

### Environnement sanitaire scolaire, menstruation et scolarité

Environ 54,6% des élèves ont connu des menstruations inattendues à l'école, ce qui a conduit 79,1% d'entre elles à retourner à la maison, principalement en raison des mauvaises conditions d'hygiène scolaire. Seules 11,0%, des élèves ont pu changer des absorbants à l'école, principalement en raison de toilettes inappropriées (50,8%) et de l'absence d'intimité (30,7%). Près de 34,9% des élèves ont manqué l'école à cause des menstruations, principalement pour douleurs et malaises (37,3%). Les besoins prioritaires incluent un kit sanitaire (71,1%) et des paracétamols (9,4%) (Tableau 5).

**Tableau 5:** environnement sanitaire scolaire, menstruation et scolarité

Variables	Privée n=302 (%)	Publique n=251 (%)	Total n=553 (%)
**Survenue inattendue des menstruations à l’école**			
Oui	164 (54,3)	138 (55,0)	302 (54,6)
Non	138 (45,7)	113 (45,0)	251 (45,4)
**Si oui, attitudes prises**	**n=164**	**n=138**	**n=302**
Retour à la maison	123 (75,0)	116 (84,1)	239 (79,1)
Récupération de l’absorbant à la direction	15 (9,2)	6 (4,4)	21 (7,0)
Voir l´infirmière	3 (1,8)	4 (2,9)	7 (2,3)
Autres à spécifier	23 (14,0)	12 (8,7)	35 (11,6)
**Changement d’absorbants à l’école**			
Oui	27 (8,9)	34 (13,6)	61 (11,0)
Non	275 (91,1)	217 (86,4)	492 (89,0)
**Raisons de non changement à l’école**	**n=275**	**n=217**	**n=492**
Toilettes pas propres	141 (51,3)	109 (50,2)	250 (50,8)
Pas des portes ou verrous des toilettes	89 (32,4)	62 (28,6)	151 (30,7)
Pas d’eau courante	20 (7,3)	22 (10,1)	42 (8,5)
Pas assez de tissus absorbants	5 (1,8)	5 (2,3)	10 (2,0)
Autres	20 (7,3)	19 (8,8)	39 (7,9)
**Absence à l’école pendant les menstruations**			
Oui	105 (34,8)	88 (35,1)	193 (34,9)
Non	197 (65,2)	163 (64,9)	360 (65,1)
**Raisons d’absence**	**n=105**	**n=88**	**n=193**
Douleur et malaises	44 (41,9)	28 (31,8)	72 (37,3)
Peur de tacher les habits	25 (23,8)	21 (23,9)	46 (23,8)
Peur des moqueries	17 (16,2)	11 (12,5)	28 (14,5)
Absence d’espaces intimes pour changer l’absorbant	5 (4,8)	7 (8,0)	12 (6,2)
Pas d’absorbant	2 (1,9)	1 (1,1)	3 (1,6)
Endroit pour jeter absorbant usé	3 (2,9)	6 (6,8)	9 (4,7)
Autres	9 (8,6)	14 (15,9)	23 (11,9)
**Besoins prioritaires pour l’HM à l’école**			
Kit sanitaire	220 (72,9)	173(68,9)	393 (71,1)
Paracétamol	35 (11,6)	17 (6,8)	52 (9,4)
Savons	13 (4,3)	14 (5,6)	27 (4,9)
Incinérateur	2 (0,7)	12 (4,8)	14 (2,5)
Eau courante	7 (2,3)	6 (2,4)	13 (2,4)
Sous-vêtement de change	4 (1,3)	8 (3,2)	12 (2,2)
Bassinet	6 (2,0)	4 (1,6)	10 (1,8)
Porte avec verrous	15 (5,0)	17 (6,8)	32 (5,8)

### Facteurs associés aux bonnes connaissances de la menstruation et de l'hygiène menstruelle

L'analyse multivariée a montré des facteurs clés associés à une meilleure connaissance des menstruations et de l'HM chez les adolescentes. Résider dans la commune de Makiso et suivre un cours d'éducation abordant l'HM se sont révélés être les principaux prédicteurs significatifs, avec respectivement (ORa=1,8; IC95%=1,0-3,4; p=0,049) et (ORa=2,7; IC95%=1,1-6,3; p=0,025). De même, les adolescentes en terminal avaient une probabilité accrue de bonne connaissance par rapport à celles des niveaux inférieurs (ORa=2,4; IC95%=1,1-5,3; p=0,025). Recevoir des informations des mères ou sœurs, s'est également révélé un facteur favorable (ORa=2,7; IC95%=1,4-5,1; p=0,002). En revanche, des variables telles que l'âge, la résidence et l'appartenance institutionnelle n'ont pas montré d'association significative après ajustement (Tableau 6).

**Tableau 6:** facteurs associés aux bonnes connaissances des menstruations et HM

Connaissance des menstruations et HM	OR [IC95]	p-value*	ORa [IC95]	p-value*
**Appartenance institutionnelle**				
Publique	Réf.			
Privée	1,0 [0,6-1,8]	0,932		
**Lieu de résidence**				
Makiso	1,9[1,0-3,4]	0,023	1,8[1,0-3,4]	0,049
Kabondo	0,6[0,1-1,8]	0,329		
Kisangani	0,5[0,1-2,2]	0,402		
Lubunga	1,4 [0,4-4,4]	0,519		
Mangobo	Réf			
Tshopo	1,2 [0,6-2,3]	0,505		
**Age**				
12 à 14 ans	0,5 [0,2-0,9]	0,017	0,8[0,4-1,7]	0,668
15 à 17 ans	Réf			
≥ 18 ans	2,4[1,2-4,4]	0,002	1,0[0,5-2,2]	0,953
**Niveau d’étude adolescente**				
Enseignement de base	0,4[0,2-0,9]	0,016	0,8[0,3-2,1]	0,700
Moyen	Réf			
Terminal	3,1 [1,7-5,5]	0,000	2,4[1,1-5,3]	0,025
**Cours abordant l’aspect de l’HM**				
Oui	2,1 [1,1-4,5]	0,033	2,7 [1,1-6,3]	0,025
Non	Réf.			
**Sources d’information**				
Mère et sœurs	2,6[1,3-4,9]	0,002	2,7[1,4-5,1]	0,002
Autres	Réf			

* Seuil de signification p<0,05

### Facteurs associés aux bonnes pratiques de l'hygiène menstruelle

L'analyse multivariée a identifié des facteurs significativement associés à une meilleure gestion de l'HM. Les adolescentes inscrites dans des écoles privées étaient plus susceptibles d'adopter de bonnes pratiques par rapport à celles des écoles publiques (ORa=0,6; IC95%= 0,3-0,9; p= 0,041). De même, un niveau d'étude universitaire des mères constituait un facteur favorable (ORa=1,9; IC95%=1,1-3,3 ; p=0,016). L'information sur les menstruations avant la ménarche augmentait également les bonnes pratiques (ORa=0,1; IC95%=0,0-0,9; p=0,047). (Tableau 7).

**Tableau 7:** facteurs associés aux bonnes pratiques de l'hygiène menstruelle

Variables indépendantes	OR [IC95]	p-value*	ORa [IC95]	p- value*
**Appartenance**				
Publique	Réf.			
Privée	0,6 [0,3-1,0]	0,041	0,6 [0,3-0,9]	0,041
**Age**				
12 à 14 ans	1,2[0,7-2,1]	0,434		
15 à 17 ans	Réf			
≥ 18 ans	1,1 [0,5-2,1]	0,784		
**Niveau d’étude**				
Base	0,9 [0,5-1,8]	0,962		
Moyen	Réf			
Terminal	0,9 [0,5-1,7]	0,691		
**Niveau d’étude de mère**				
Primaire	1,2[0,3-3,7]	0,742		
Secondaire	Réf			
Universitaire	1,8[1,03-3,1]	0,023	1,9[1,1-3,3]	0,016
**Informer avant ménarche**				
Oui	0,1 [0,0-0]	0,027	0,1[0,0-0,9]	0,047
Non	Réf.			

* Seuil de signification p<0,05

## Discussion

Cette étude a porté sur les connaissances, attitudes et pratiques des élèves adolescentes sur l'HM à Kisangani. Ces résultats ont montré que les prévalences des bonnes connaissances et pratiques des élèves adolescentes étaient très faibles.

### Connaissances sur la menstruation et l'hygiène menstruelle

Dans cette étude, 92% des participantes étaient informées de la menstruation avant leurs ménarche. Ce résultat est similaire à celui observée à Goma [12] mais diffère de celui de Lubumbashi [13]. Cette différence pourrait s'expliquer par les variations culturelles entre les régions Est et Sud-Est de la RDC. Les principales sources d'information étaient les mères et les sœurs, confirmant le silence culturel persistant autour des menstruations en RDC [9]. En revanche, en Gambie, les enseignants jouent un rôle plus important dans l'éducation menstruelle [1].

Cependant, en dépit d'être informée avant la ménarche, la majorité des filles avaient des connaissances erronées concernant les causes et origines des menstruations. Par contre, 92% de filles connaissaient les types d'absorbant appropriés à utiliser pendant les menstrues. Cette situation souligne la faible qualité d'informations que les adolescentes reçoivent avant leurs ménarche, qui se limiterait aux différents types d'absorbants à utiliser sans une explication détaillée sur les menstruations.

La prévalence de bonnes connaissances sur la menstruation et l'HM était de 11,0% dans cette étude, bien inférieur aux 38,9% observé à Goma [12]. Au-delà des différences méthodologiques, cette disparité pourrait s'expliquer par la présence de plusieurs organisations non gouvernementales (ONG) humanitaires à Goma, qui, dans le contexte des conflits armés, sensibilisent les adolescentes sur la lutte contre les violences sexuelles et abordent parfois les questions d'HM.

### Attitudes et pratiques de l'hygiène menstruelle

L'étude a montré que 81,6% des participantes avaient une attitude défavorable à la ménarche. Ce résultat, similaire à celui observé à Lubumbashi et en Gambie [1], remettent en cause la qualité des informations reçues avant la ménarche.

Par ailleurs, 82% des participantes ont utilisé des absorbants appropriés lors de leurs dernières menstruations. Ce résultat est comparable à celui observée à Goma [12] mais supérieur à celui observée en Gambie [1], probablement en raison des différences entre les contextes d'étude qui conditionnent l'accès aux produits absorbants: urbain en RDC et rural en Gambie.

Cependant, seulement 41,2% des participantes changeaient les produits absorbants au moins trois fois par jour. Ce résultat est similaire à celui observée en Gambie [1] mais diffère de ceux observés à Goma [12] et en Ouganda [6]. Cette différence semble liée à des disparités socio-économiques entre les contextes d'étude qui conditionnent l'accès à des produits absorbant en quantité suffisante.

Quant au séchage des tissus absorbants réutilisables, la majorité des participants le faisaient à l'abri des regards, soit à l'extérieur et au soleil (51,7%). Ces résultats sont presque similaires à ceux observés à Goma [12] et supérieurs à ceux observés en Gambie [1]. En dépit de ces différences, ces résultats seraient le reflet du tabou persistant autour des menstruations dans les sociétés africaines. Concernant l'élimination des tissus absorbants à usage unique, 62,4% des participantes les faisaient de façon inadéquate. Cette pratique a des impacts environnementaux négatifs, tels que le blocage des égouts et la perturbation de la microflore du sol [14].

La prévalence de bonnes pratiques de l'HM était de 12%. Ce résultat est similaire à celui trouvé en Ouganda [6], mais inférieur à ceux observés à Goma et en Éthiopie [12,15]. Les différences méthodologiques dans l'estimation des pratiques de l'HM expliqueraient ces variations. Ce résultat pourrait s'expliquer par la mauvaise qualité de l'information, le tabou persistant sur les menstruations et l'absence d'interventions adaptées pour améliorer les pratiques d'HM.

### Environnement sanitaire scolaire, menstruation et scolarité

Plus de la moitié des enquêtées (54,6%) ont vécu l'apparition inattendue des menstrues à l'école, obligeant 79,1% d'entre elles à rentrer à la maison, principalement en raison des mauvaises conditions d'hygiène scolaire. Par ailleurs, 34,9% des participantes ont manqué des cours suite à la menstruation. Les raisons évoquées, notamment les douleurs et/ou malaises, la peur de tacher les habits et l'absence des toilettes intimes, étaient similaires celles trouvées en Éthiopie [15]. Ces résultats soulignent l'urgence d'améliorer les conditions d'HM en milieu scolaire pour promouvoir l'égalité de sexe et l'équité.

### Facteurs associés à la gestion de l'hygiène menstruelle

L'analyse multivariée a montré que le fait de résider dans la commune de Makiso, de suivre les cours de l'éducation à la vie qui intègre les notions de l'HM, le niveau terminal et l'influence des mères ou sœurs comme sources d'information sur la menstruation comme des prédicteurs significatifs d'une bonne connaissance sur la menstruation et l'HM. Sommer *et al*. ont montré que l'intégration de l'HM dans les programme scolaire améliore les connaissances de l'HM des adolescentes [16]. Les études menées en Éthiopie et au Nigeria ont montré que le niveau terminal influençait les connaissances sur l'HM [17,18]. Ces résultats suggèrent que des interventions sur les programmes du système éducatif et la sensibilisation des mères ou sœurs sur l'HM vont contribuer à l'amélioration des connaissances des adolescentes sur la menstruation et l'HM.

Quant aux bonnes pratiques de l'HM, l'analyse multivariée a montré que la scolarisation dans les écoles privées, l'éducation parentale, le niveau universitaire de la mère et avoir l'information sur la menstruation avant la ménarche étaient significativement associés à la bonne pratique de l'HM. Ces résultats corroborent ceux des autres études menées ailleurs qui montrent que la préparation avant la ménarche permet aux jeunes filles de mieux comprendre et gérer leur santé menstruelle [18,19]. Celles qui étaient scolarisées dans les écoles privées [16] ou ayant des mères instruites avaient une plus grande probabilité d'adopter de bonnes pratiques [18,20].

### Limites et perspectives

Cette étude présente des limites. En effet, il est possible que le biais de désirabilité sociale ait pu affecter les réponses des participantes sur l'HM. Ce biais a été atténue par le briefing des participantes et l'utilisation des enquêtrices sages-femmes. De plus l'étude a exclu les adolescents non scolarisés, offrant ainsi une vue partielle de la réalité. Enfin, l'étude était quantitative et n'a pas exploré les raisons des attitudes et pratiques observées, d'où la nécessité d'une étude qualitative ultérieure.

### Recommandations et implications pratiques

Les familles, principalement les mères et sœurs, sont les principales sources d'informations sur l'HM, mais la qualité de ces informations nécessite d'être améliorée par le renforcement des capacités des membres des familles, notamment les mères et les sœurs. Aussi, il est essentiel d'améliorer le cours d'éducation à la vie familiale avec un contenu structuré et de renforcer les capacités des enseignants. Par ailleurs, les mauvaises conditions d'eau, d'hygiène et d'assainissement dans les écoles constituées les barrières à la bonne pratique de l'HM chez les adolescentes scolarisées. Ceci implique la nécessité de l'amélioration des infrastructures scolaires en prenant en compte ces aspects de l'HM.

## Conclusion

Cette étude révèle des lacunes importantes dans les connaissances, attitudes et pratiques en matière d'HM chez les adolescentes scolarisées à Kisangani. Nos résultats suggèrent des déficiences significatives dans la qualité de l'information fournie aux adolescentes, principalement par les mères et les grandes sœurs. A ce déficit de l'information de qualité s'ajoutent les mauvaises conditions d'hygiène dans les écoles qui ne facilitent pas une bonne gestion de l'HM dans les écoles et entraîne des absences scolaires. Face à ces défis, il est impératif d'améliorer la qualité des messages provenant de sources éducatives et d'améliorer les conditions d'eau, d'hygiène et d'assainissement dans les écoles.

### Etat des connaissances sur le sujet

- La menstruation génère des besoins spécifiques en matière d'HM, incluant l'accès aux informations adéquates, produits absorbants appropriés et installations sanitaires privées;
- La gestion de l'HM demeure un problème de santé publique en Afrique subsaharienne à cause de l'influence négative de divers facteurs socio-culturels, économiques et physiques;
- Les études sur la gestion de l'HM chez les élèves adolescentes du secondaire sont limitées en RDC et particulièrement dans la province de la Tshopo.

### Contribution de notre étude à la connaissance

- Les connaissances, attitudes et pratiques des élèves adolescentes de la ville de Kisangani (RDC) demeurent encore largement sous-optimales;
- Le niveau d'instruction universitaire de la mère et le fait d'avoir reçu l'information sur les menstruations avant la ménarche sont susceptibles d'améliorer la prévalence de bonnes pratiques de l'HM chez les élèves adolescentes;
- L'environnement scolaire malsain influence négativement la gestion de l'HM chez les adolescentes et entrainent des absences à l'école.

## Remerciements

Nous remercions tous ceux qui nous ont facilité la réalisation de ce travail : les sages-femmes Batshi Ndjangosi Rose et Eunice Bolota Baseangandi pour collecter les données auprès des élèves, les responsables des écoles pour la collaboration et l'autorisation pour mener la recherche et Blaise Lisungi et Benoit pour l'encodage des données.
